# Reef Fishes in Biodiversity Hotspots Are at Greatest Risk from Loss of Coral Species

**DOI:** 10.1371/journal.pone.0124054

**Published:** 2015-05-13

**Authors:** Sally J. Holbrook, Russell J. Schmitt, Vanessa Messmer, Andrew J. Brooks, Maya Srinivasan, Philip L. Munday, Geoffrey P. Jones

**Affiliations:** 1 Department of Ecology, Evolution and Marine Biology, University of California Santa Barbara, Santa Barbara, California, 93106, United States of America; 2 Coastal Research Center, Marine Science Institute, University of California Santa Barbara, Santa Barbara, California, 93106, United States of America; 3 Australian Research Council Centre of Excellence for Coral Reef Studies, James Cook University, Townsville, Queensland, 4811, Australia; 4 School of Marine and Tropical Biology, James Cook University, Townsville, Queensland, 4811, Australia; Leibniz Center for Tropical Marine Ecology, GERMANY

## Abstract

Coral reef ecosystems are under a variety of threats from global change and anthropogenic disturbances that are reducing the number and type of coral species on reefs. Coral reefs support upwards of one third of all marine species of fish, so the loss of coral habitat may have substantial consequences to local fish diversity. We posit that the effects of habitat degradation will be most severe in coral regions with highest biodiversity of fishes due to greater specialization by fishes for particular coral habitats. Our novel approach to this important but untested hypothesis was to conduct the same field experiment at three geographic locations across the Indo-Pacific biodiversity gradient (Papua New Guinea; Great Barrier Reef, Australia; French Polynesia). Specifically, we experimentally explored whether the response of local fish communities to identical changes in diversity of habitat-providing corals was independent of the size of the regional species pool of fishes. We found that the proportional reduction (sensitivity) in fish biodiversity to loss of coral diversity was greater for regions with larger background species pools, reflecting variation in the degree of habitat specialization of fishes across the Indo-Pacific diversity gradient. This result implies that habitat-associated fish in diversity hotspots are at greater risk of local extinction to a given loss of habitat diversity compared to regions with lower species richness. This mechanism, related to the positive relationship between habitat specialization and regional biodiversity, and the elevated extinction risk this poses for biodiversity hotspots, may apply to species in other types of ecosystems.

## Introduction

Environmental drivers associated with climate change, as well as other human and natural disturbances, are expected to result in the loss of biodiversity in a variety of ecosystems [1,2,3], underscoring the need to fully understand the link between biodiversity and ecosystem function (BEF). Seminal experiments that manipulated species richness of primary producers in terrestrial, freshwater and, to a lesser degree, marine reef ecosystems have shown that ecosystem functions such as biomass production, resource use, and nutrient cycling are often strongly influenced by changes in producer biodiversity [4–7]. Similarly, in systems where ‘top-down’ control is strong, such as in many marine reef ecosystems [8], changes in biodiversity of consumers can greatly influence these and other rate processes [9]. What we know comparatively little about is how changing biodiversity of foundation taxa will influence their habitat-providing function, and thus the biodiversity of associated species [10,11]. This is a particularly critical issue when the foundation taxa support a high diversity of iconic species, as is the case with corals and fishes [12].

The few studies that have experimentally manipulated the richness of habitat-providing aquatic and terrestrial plants have shown inconsistent effects on the biodiversity of the associated animal communities [9–11,13]. It has been suggested that the general lack of strong effects of aquatic macrophyte richness might be due to a low degree of habitat specialization in the systems studied [9]. The effects of habitat degradation will likely be exacerbated in regions where there are strong species-specific associations between mobile organisms and sedentary habitat-providing species that make up the underlying habitat. However, to date there have been no rigorous tests of this important hypothesis such as identical experiments that are repeated over global gradients of species diversity and specialization.

Compared to most other marine ecosystems, many species in coral reef fish communities exhibit high levels of habitat specialization, which may make these communities especially vulnerable to reductions in species richness of habitat-providing corals [14,15]. Coral reefs support the greatest biodiversity of all marine ecosystems, reflecting in part the complex habitat provided by reef-forming corals [16–19]. This biodiversity is at risk as coral reefs are highly threatened by global change and anthropogenic disturbances [2,18,20–24]. Warming, ocean acidification, altered water quality and other environmental changes are forecast to reduce the number and diversity of corals on reefs in the future [24–27].

Since many of the effects of global change and other perturbations on coral reef communities will be mediated through impacts on habitat-providing corals, there is an urgent need to understand how loss of habitat diversity in this ecosystem will affect associated organisms [12,28]. This is particularly the case for fishes due to the singular importance of coral reefs to their global biodiversity. Although coral reefs cover much less than one percent of the ocean floor, they support between a quarter and a third of all species of marine fish [29]. This diversity of fishes is not uniformly distributed among coral reef regions of the world with, for example, a strong geographic gradient in biodiversity of both corals and reef fishes from east to west across the Indo-Pacific culminating in the Coral Triangle diversity hotspot [30–32]. Exploration of the consequences of such variation in regional diversity to the response of fishes to habitat degradation has shown inconsistent results [28], ranging from great loss of fish biodiversity [33] to comparative insensitivity [34]. Because these studies were done using different methods in geographic regions with differing background species pools, it has not been possible to evaluate whether inconsistent findings reflect variation in methodology or systematic differences in attributes of the fish assemblages. We posit that the effect of reduced coral diversity on fish species richness should increase with the degree of habitat specialization within a regional fish community [14]. By extension, if mean habitat specialization co-varies positively with the size of the regional species pool, which may often be the case [35,36], then so will the effect size for the same loss of habitat diversity.

**Fig 1:**
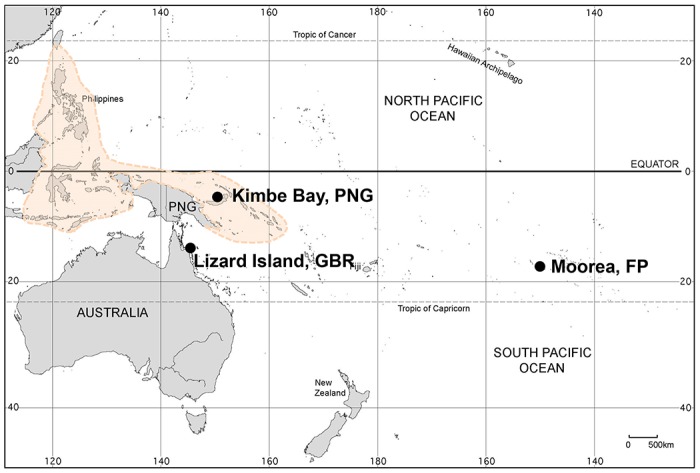
Map of the Indo-Pacific region showing the locations of the three study sites. The area shaded in color delineates the Coral Triangle biodiversity hotspot. Map modified from the U.S. CIA Oceania physical map (https://www.cia.gov/library/publications/the-world-factbook/index.html) and is for representative purposes only.

**Fig 2:**
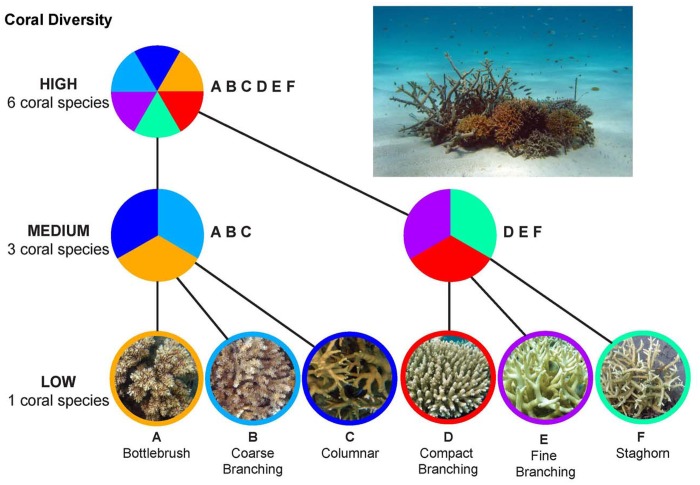
Schematic diagram of the experimental design. At each of the three study sites, replicate 1 m dia patch reefs were constructed on sandy bottom using the same set of 6 coral morphotypes (bottom row pictures) to explore how reductions in habitat diversity of a patch (holding coral cover constant) affected the biodiversity of associated fishes. There were 3 levels of habitat diversity: the high diversity treatment (top) were patch reefs that contained equal amounts of all 6 coral species, the two medium diversity treatments consisted of two different combinations of 3 coral species (middle row), and the six low diversity treatments consisted of each coral species alone (bottom row). There were 5 replicates of each of the 9 treatments. The 6 coral morphotypes were: (A) Bottlebrush, (B) Coarse branching, (C) Columnar, (D) Compact branching, (E) Fine branching and (F) Staghorn (see Table 1). The inset image is a picture of a high diversity treatment plot at Lizard Island (photo credit: inset & corals A-C, E-F: V. Messmer; coral D: M. Bonin).

Habitat degradation already is occurring on coral reefs [19–22] and Global Climate Change (GCC) and Ocean Acidification (OA) are predicted to have further negative impacts on habitat-providing corals through increased intensity of storms, temperature excursions above thermal bleaching tolerances, and an impaired capacity to calcify [23]. Initial projections of a complete loss of corals from these drivers have been replaced by a more nuanced scenario in which future coral reefs will be comprised of a smaller subset of corals that have been described as ‘winners’ [24–27,37]. While the likely attributes of corals able to cope in a warmer, more acidic ocean in the future is an area of active research, the general consensus is that there will be a loss of coral diversity. We estimated the sensitivity of local fish communities to changes in the richness of habitat-forming coral morphotypes, as a function of the regional species pool of fishes, by conducting an identical field experiment at each of 3 geographic locations along the Indo-Pacific diversity gradient (Fig 1). Kimbe Bay, Papua New Guinea (PNG) is in the Coral Triangle biodiversity hotspot and has the greatest species richness of fishes (ca. 1600 species in PNG [31]), whereas Moorea, French Polynesia, located in the central South Pacific has the lowest (French Polynesia has less than half of the species richness of reef fish in PNG [31]). Lizard Island on the Great Barrier Reef, Australia has a species pool somewhat lower than Kimbe Bay (northern GBR ca. 10–15 percent lower than PNG [30]). The experimental design (Fig 2, Table 1) simulated the same level of patch reef scale variation in coral (habitat) richness across these three localities. Our experiments revealed how and why the same amount of habitat degradation can result in systematically different biodiversity responses in communities across a geographic diversity gradient.

**Table 1:** Coral species for each of the 6 habitat morphotypes used in the experiment at each of the 3 localities.

Morphotype	MOOREA	LIZARD ISLAND	KIMBE BAY
**Low Diversity**			
**A** Bottlebrush	*Acropora elseyi*	*Acropora loripes*	*Acropora carduus*
**B** Coarse branching	*Pocillopora verrucosa*	*Pocillopora damicornis*	*Pocillopora damicornis*
**C** Columnar	*Porites rus*	*Porites cylindrica*	*Porites cylindrica*
**D** Compact branching	*Pocillopora eydouxi*	*Acropora nasuta*	*Acropora nasuta*
**E** Fine branching	*Acropora fragile*	*Seriotopora histrix*	*Seriotopora histrix*
**F** Staghorn	*Acropora pulchra*	*Acropora muricata*	*Acropora grandis*
**Medium Diversity 1:**	A + B + C		
**Medium Diversity 2:**	D + E + F		
**High Diversity:**	A + B + C + D + E + F		

## Results and Discussion

**Fig 3:**
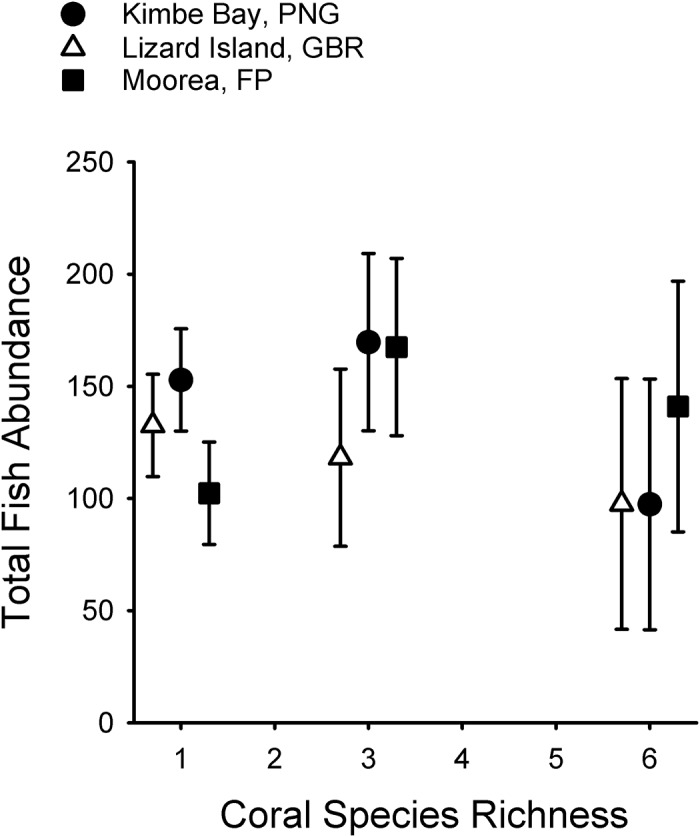
Total number of fish individuals per experimental patch reef on each coral diversity treatment at the final census at the three study sites. Data are the mean (± 1 SE) number of individuals per plot. For clarity, placement of symbols corresponding to coral species richness values for Lizard Island, GBR (open triangles) and Moorea, FP (filled squares) have been shifted slightly along the x-axis. N = 30 patch reefs per geographic location for coral species richness of 1 species, N = 10 patch reefs per geographic location for coral species richness of 3 species, and N = 5 patch reefs per geographic location for coral species richness of 6 species.

Initial analyses explored overall patterns of fish abundance among geographic locations and among coral diversity levels. The level of coral diversity had no effect on the abundance of fishes on a patch reef (F_2,130_ = 0.60; P > 0.55; locations pooled), nor was there any difference in the mean abundance of fishes per patch reef among the three geographic locations (F_2,130_ = 0.73; P > 0.48; coral diversity treatments pooled). The mean number of fish per m^2^ (± 1 SE) was 150.4 (± 22.9) at Kimbe, 125.5 (± 14.3) at Lizard and 121.1 (± 17.1) at Moorea. The similarity in abundance both among treatments and locations (Fig 3) indicates that any differences observed in species richness of fishes cannot be explained by differences in the numbers of individual fish present on the experimental patch reefs.

**Fig 4:**
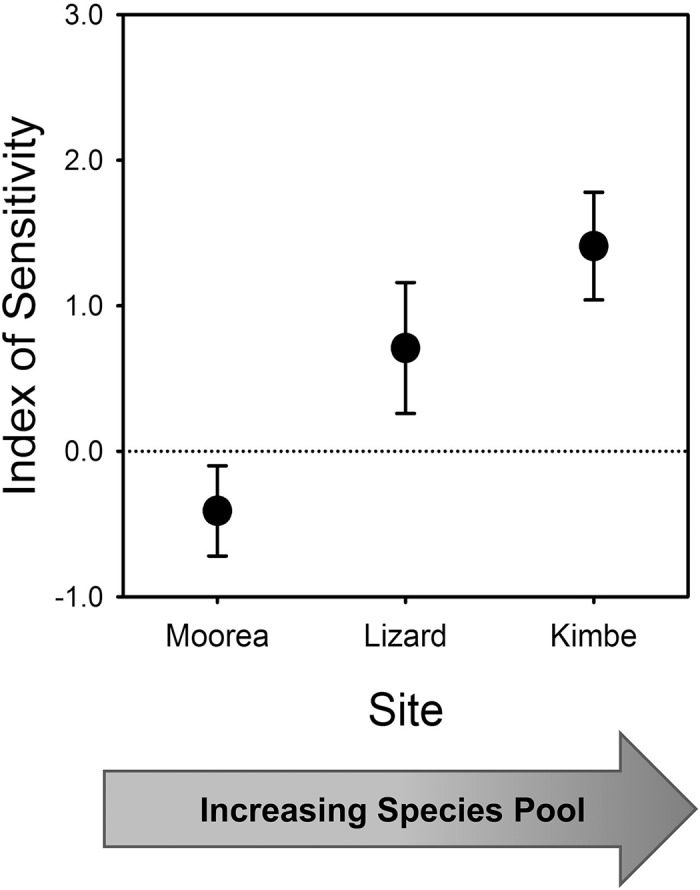
Sensitivity of coral reef fish species richness at the three study locations to the same reduction in coral (habitat) diversity. The Index of Sensitivity is the slope (± 1 SE) of the relationship between the number of coral species on an experimental patch reef and the number of species of fishes at the final survey (see S1 Fig). Greater positive values indicate proportionately greater declines in species richness of fish for the same reduction in coral (habitat) diversity, and the dashed line at 0 denotes no difference in species richness of fish over the range in coral diversity used in the experiment. Each slope estimate is based on N = 45 patch reefs.

By contrast with abundance, the relationship between variation in coral diversity and species richness of fishes differed substantially among geographic locations (coral diversity x location interaction: F_4,126_ = 2.91; P < 0.025; S1 Fig). The slopes of the coral diversity—fish richness relationship differed, which indicates that the proportionate decline (sensitivity) in fish species richness to the same reduction in coral diversity varied markedly across the geographic gradient (Fig 4). Moorea showed no sensitivity and Kimbe the most to the same variation in local coral diversity (Fig 4). The sensitivity ranking mirrored the size of the regional species pool of fishes among these geographic locations, indicating that the proportionate loss in biodiversity of fishes to lessening of coral diversity scaled positively with the size of the pool.

**Fig 5:**
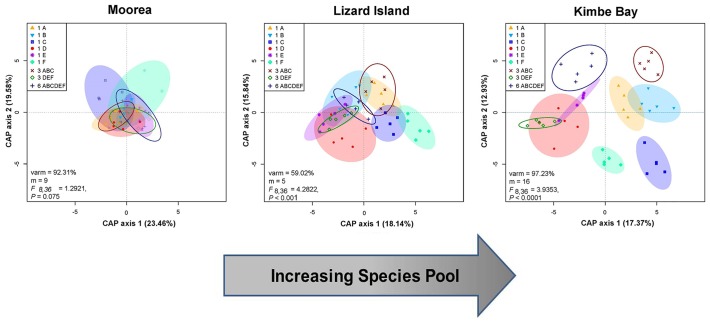
Canonical analysis of principal coordinates (CAP) ordination plot (Bray-Curtis) of fish assemblage data for each experimental treatment at each geographic location. Each point represents a separate patch reef, and the (color-coded) shaded clusters are dispersion ellipses for the 6 different single coral (low habitat diversity) treatments. The outlined dispersion ellipses represent the confidence limits for the 3-species (medium) and 6-species (high) treatments. N = 5 replicate patch reefs for each of the 9 coral diversity treatments at each geographic location (see Fig 2). The CAP analyses captured a large amount of the variation in community structure in the first two components, with the two primary axes (CAP 1 and CAP 2) accounting for 43% (Moorea), 34% (Lizard Island) and 30% (Kimbe Bay) of the total variance. Dispersion ellipses are based on 0.9 confidence limits of the standard deviation of point scores. CAP groupings were strongly supported, and results of Multivariate Analyses of Variance (MANOVA) (given at the bottom of each panel) constructed on Bray-Curtis dissimilarity matrices of log-transformed fish abundance data revealed that fish communities differed significantly among the treatments at Lizard Island and Kimbe Bay but not at Moorea.

Canonical analysis of principal coordinates (CAP) revealed that the geographic pattern in sensitivity of fish communities to loss of coral diversity was due to marked differences in the degree of habitat specialization among the fish communities (Fig 5). Fish assemblages were very distinct among the 6 coral species at Kimbe, as evidenced by the very limited overlap in the dispersion ellipses of the low diversity (i.e., single coral species) treatments (Fig 5), a pattern driven largely by the non-overlapping distributions of several species within the highly specialized genera of coral gobies, *Gobiodon* and *Paragobiodon*, and the restriction of the obligate corallivore, *Chaetodon baronessa*, to plots containing either Pocilloporid or Acroporid corals. By contrast, fish assemblages were remarkably similar among the same experimental suite of coral morphotypes at Moorea (i.e., high overlap in dispersion ellipses), with differences among treatments reflecting differences in the abundance of several Pomacentrid species (*Chromis viridis*, *Dascyllus flavicaudus*, and *Pomacentrus pavo*). Separation in dispersion ellipses at Lizard Island was intermediate between Kimbe and Moorea (Fig 5) and was largely determined by non-overlapping distribution patterns of *Gobiodon citrinus* and *Paragobiodon xanthosomus* as well as *Chromis viridis* and *Dascyllus aruanus*. Dominant species-specific loading scores on the two CAP axes are presented in S2–S4 Tables. Multivariate analyses of variance (MANOVA) indicated that the species composition of the fish assemblage was statistically different among the treatments at Kimbe (F_8,36_ = 3.93; P < 0.001) and Lizard (F_8,36_ = 4.28; P < 0.001), but not at Moorea (F_8,36_ = 1.29; P = 0.075). Thus the degree of habitat specialization within a fish community increased with increases in the regional species pool of fishes.

Our results imply that the relationship between biodiversity and the habitat-providing function of corals varies predictably among communities that differ in mean habitat specialization, which itself generally tends to scale positively with the size of the regional species pool [35]. As a consequence, habitat-associated species in diversity hotspots are at greater risk of local extinction to a given loss of habitat diversity compared to regions with lower species richness. This geographic pattern mirrors studies on high diversity reefs where loss of coral resulted in disproportionately greater losses of habitat specialists than generalists [14,15,38]. This is further supported by field and laboratory studies that have shown inflexibility in use of corals by fish that are coral specialists [39–42]. Given the strong link between the degree of specialization and large-scale diversity gradients [35], this mechanism that exacerbates extinction risk in biodiversity hotspots may be a general phenomenon.

Two additional factors may contribute to a higher risk of local extinction where organisms use a narrow range of habitats. First, specialists often face a “double jeopardy”, not only because of their susceptibility to loss of habitat, but also because they often have small population sizes that inherently are more vulnerable [14]. Of course, a wide geographic distribution and/or high levels of connectivity among populations can help species offset their risk of extinction [43,44]. Coral reef fishes may also face greater extinction risk in diversity hotspots than elsewhere because many perturbations and drivers associated with GCC and OA are predicted to reduce both coral diversity and coral abundance [19–27]. Second, co-occurring coral species differ greatly in the biodiversity of fishes and other species they support [28,45], as well as in their vulnerability to disturbances and drivers associated with GCC and OA [24–27]. If corals with greater habitat-providing functions have higher risk of local extinction, the knock-on effect on biodiversity of coral-associated species will be greater in diversity hotspots than elsewhere due to greater habitat specificity. These same arguments imply that communities in regions of lower diversity—with proportionately fewer habitat specialists—will be more resistant to reductions in the diversity of coral habitat.

## Methods

### Field experiment

To test the impact of the regional species pool on the coral-fish diversity relationship, the same experiment was conducted in lagoons of Schumann Island in Kimbe Bay, Papua New Guinea (5°31’S, 150°5’E), Lizard Island on the Great Barrier Reef, Australia (14° 41’S, 145° 27’E), and Moorea in French Polynesia (17° 30’S, 149° 50’W) (Fig 1). These locations occupy different positions along the Indo-Pacific diversity gradient and vary in the sizes of their background species pools of fishes (Fig 1). In each location, 45 patch reefs were constructed using six abundant, co-occurring coral species that were major habitat providers for fish; in all, six different coral morphotypes were represented (Table 1). Coral morphotypes were represented by species that were matched morphologically across the three locations. Individual patch reefs were composed of one, three or all six of the coral species. There were six low diversity (single coral species) treatments, two medium diversity treatments (two different combinations of three coral species), and 1 high diversity treatment (all six coral species), with 5 replicates of each treatment (Table 1). The species of corals used in the experiment were selected to include species that exhibit a broad range of structural morphologies and potential sensitivities to climate change and other environmental stressors [14,17,25,38,40,41,45].

Patch reefs, each 1 m in diameter and 0.5 m high, were built at 3 to 7 m depth on large flat sandy areas where no other habitat structure was present. The size of the patch reefs represented the scale at which the fish species of interest for the experiment typically interact with their habitat, and is the median size of naturally-occurring patch reefs in lagoons [46]. Patch reefs of this size can support a variety of types of coral, and include numerous species. Reefs were placed 15 m apart from each other and from any neighboring reef structures to minimize fish movement between reefs. The base of each patch reef consisted of dead coral rubble, which was covered with the same amount of live coral to achieve 90% live coral cover. Reefs were initially unoccupied by fish. Fish were allowed to naturally colonize over 8–12 months and the patch reefs were surveyed by scuba divers four times during the period. Divers counted individuals of all species observed on or interacting with the patch reefs. This included species resident on the plots, as well as those observed feeding or refuging on the coral. In addition to small-bodied species that resided on the patch reefs, our surveys included young stages of larger mobile reef species that use coral structure as juvenile habitat (S1 Table). Recruitment was rapid and patterns of abundance and diversity were established after 2 to 3 months. For each survey, the abundance of every fish species observed was recorded. The volume of live coral on each patch reef was assessed using photographs and diver measurements during the four surveys. Minor repairs to reefs were carried out where necessary after each survey to keep the volume of live coral constant across treatments and locations. Holding the volume of coral as constant as possible during the course of the experiment prevented over yielding due to development of higher biomass or volume of the coral habitats in the high diversity treatments, factors that could potentially affect patterns of species richness of the associated fishes [47,48].

### Statistical analyses

Estimates of fish species richness and total abundance were based on the total number of species or individuals observed on each patch reef during the final survey [49]. We first explored both the interactive and independent effects of location (Moorea, Lizard, Kimbe) and coral species richness (1, 3 or 6 species) on total abundance of fish per patch reef with a two-way ANOVA (patch reefs as replicates). There was a total of 145 patch reefs (45 per geographic location). For analyses of the effects of coral diversity these patch reefs were assigned to three levels (high coral diversity, N = 5 replicates per location, intermediate diversity, N = 10 replicates per location, and low diversity, N = 30 replicates per location). Because there was no difference in how abundance varied with coral diversity among the geographic locations (coral diversity x location interaction: F_4,126_ = 0.63; P > 0.6), we report abundance results for the reduced model. Because sample sizes were unequal among the three coral diversity levels, Type III Sums of Squares were used to determine statistical significance. We also used a two-way ANOVA (patch reefs as replicates) to test the relationship between coral diversity and species richness of fish among the three locations. Shapiro-Wilk and Levene’s tests for normality and homogeneity of variances indicated no response variable required transformation to satisfy assumptions of analysis of variance. The slope of the relationship between coral diversity and fish species richness was calculated for each geographic location by fitting linear regressions to the data for individual reefs, which provided estimates of the sensitivity of the fish communities to changes in coral diversity. All ANOVA, regression and diagnostic results were generated using SAS/STAT software PROC GLM, Version 9.2, of the SAS System for Windows.

To explore the influence of coral species richness on the composition of fish communities (i.e., species composition and relative species abundance), we used canonical analyses of principal coordinates (CAP). CAP analyses were based on the Bray-Curtis dissimilarity measure of log-transformed abundance data [ln(x+1)] of the fish on each replicate reef during the last survey. Lognormal transformations were applied to reduce the influence of highly abundant species. Newly recruited individuals of Apogonid species were excluded from all multivariate analyses and those involving total abundance of individuals because of very high settlement at Lizard Island just prior to the final survey. Extremely rare species (total number sighted over 12 months below five individuals) also were excluded from multivariate analyses.

The number of permutations in the CAP analyses was set to 100 and the analysis was allowed to select the optimal number of meaningful PCO axes (*m*) required to provide the best distinction between groups, maximize the proportion of correct allocations to the grouping variable, and minimize misclassification error. The first two axes, which explained most of the variation, were used to construct ordination plots. Analyses and plots were performed using the R [50] statistical packages vegan [51], BiodiversityR [52], MASS [53] and mvpart [50].

This study was approved by the James Cook University Animal Ethics Committee (AEC, Approval No. A1207) and the University of California Santa Barbara Institutional Animal Care and Use Committee (IACUC, Protocol 639). Permits to construct patch reefs were issued by the Great Barrier Reef Marine Park Authority (Permit No. G07/21637.1, Lizard Island), Haut-commissariat de la République en Polynésie Française (DRRT) (Protocole d’Accueil 2006–2007, Moorea) and permission from Schuman Island elders (PNG).

## Supporting Information

S1 Table Species list for fishes observed at Kimbe Bay, Lizard Island, and Moorea. (DOCX)

S2 Table Species loading scores obtained from a Canonical Analysis of Principal Coordinates (CAP) ordination plot constructed on a Bray-Curtis dissimilarity matrix of log-transformed fish abundance data collected from Moorea, French Polynesia. The CAP analysis examining the fish communities present on each of the 45 1 m^2^ experimental plots captured a large amount of the variation in community structure in the first two components, with the two primary axes (CAP 1 and CAP 2) accounting for 43% of the total variance. Only those species with loadings scores < -0.2 or > 0.2 on at least one of the two axes (29 out of 57 species observed) are presented.
(DOCX)

S3 Table Species loading scores obtained from a Canonical Analysis of Principal Coordinates (CAP) ordination plot constructed on a Bray-Curtis dissimilarity matrix of log-transformed fish abundance data collected from Lizard Island, Great Barrier Reef, Australia. The CAP analysis examining the fish communities present on each of the 45 1 m^2^ experimental plots captured a large amount of the variation in community structure in the first two components, with the two primary axes (CAP 1 and CAP 2) accounting for 34% of the total variance. Only those species with loadings scores < -0.2 or > 0.2 on at least one of the two axes (65 out of 107 species observed) are presented.
(DOCX)

S4 Table Species loading scores obtained from a Canonical Analysis of Principal Coordinates (CAP) ordination plot constructed on a Bray-Curtis dissimilarity matrix of log-transformed fish abundance data collected from Kimbe Bay, Papua New Guinea. The CAP analysis examining the fish communities present on each of the 45 1 m^2^ experimental plots captured a large amount of the variation in community structure in the first two components, with the two primary axes (CAP 1 and CAP 2) accounting for 30% of the total variance. Only those species with loadings scores < -0.2 or > 0.2 on at least one of the two axes (59 out of 99 species observed) are presented.
(DOCX)

S1 Fig Total number of fish species per experimental patch reef on each coral diversity treatment at the final census at the three study sites. Data are the mean (± 1 SE) number of fish species per plot. Lines represent linear regressions fitted to the individual plot data, and the slopes of these lines provide an estimate of the sensitivity of fish species richness to changes in coral diversity for each location (see Fig 4); Moorea: F_1,43_ = 1.77; P = 0.19; Lizard: F_1,43_ = 2.41; P = 0.13; Kimbe: F_1,43_ = 14.18; P < 0.001. N = 30 patch reefs per geographic location for coral diversity of 1 species, N = 10 patch reefs per geographic location for coral diversity of 3 species, and N = 5 patch reefs per geographic location for coral diversity of 6 species.
(DOCX)
