# Supplementary material for: Reef Fishes in Biodiversity Hotspots Are at Greatest Risk from Loss of Coral Species
Source: PLoS One. 2015 May 13;10(5):e0124054. doi: 10.1371/journal.pone.0124054 (PMC4430502; doi:10.1371/journal.pone.0124054)
Supplement: S1 Table — (DOCX) [file pone.0124054.s001.docx]

**S1 Table.** Species list for fishes observed at Kimbe Bay, Lizard Island, and Moorea. An X indicates a species was present on the experimental reefs at a given geographic location.

| Family | Genus species | Kimbe Bay | Lizard Island | Moorea |
| --- | --- | --- | --- | --- |
| Acanthuridae | Acanthuridae species A | X | X |  |
|  | *Acanthurus nigricauda* |  |  | X |
|  | *Acanthurus nigrofuscus* |  |  | X |
|  | *Acanthurus olivaceus* |  |  | X |
|  | *Acanthurus triostegus* |  |  | X |
|  | *Ctenochaetus binotatus* |  |  | X |
|  | *Naso annulatus* |  |  | X |
|  | *Naso literatus* |  |  | X |
|  | *Naso unicornis* |  |  | X |
|  | *Zebrasoma scopas* |  |  | X |
| Apogonidae | *Apogon leptacanthus* |  | X |  |
|  | Apogonidae species A |  | X |  |
|  | *Apogon cyanosoma* | X |  |  |
|  | *Apogon doederleini* |  | X |  |
|  | *Apogon cf. exostigma* |  | X |  |
|  | *Apogon fraenatus* | X | X |  |
|  | *Apogon fragilis* |  | X |  |
|  | *Apogon fuscus* |  | X |  |
|  | *Apogon hartzfeldii* | X |  |  |
|  | Apogonidae species B |  | X |  |
|  | *Apogon thermalis* | X | X |  |
|  | *Apogon ventrifasciatus* | X | X |  |
|  | *Cheilodipterus artus* |  | X |  |
|  | *Cheilodipterus parazonatus* |  | X |  |
|  | *Cheilodipterus quinquelineatus* | X | X |  |
|  | *Foa brachygramma* | X |  |  |
|  | *Pterapogon aff. kauderni* | X |  |  |
|  | *Ostorhinchus cyanosoma* |  | X |  |
|  | *Ostorhinchus nigrofasciatus* |  |  | X |
| Balistidae | *Balistapus undulatus* | X |  | X |
|  | *Balistoides viridescens* | X |  |  |
|  | *Odonus niger* | X |  |  |
|  | *Pseudobalistes flavimarginatus* | X | X |  |
|  | *Rhinecanthus aculeatus* | X | X | X |
|  | *Rhinecanthus rectangulus* |  |  | X |
|  | *Rhinecanthus verrucosus* | X |  |  |
|  | *Sufflamen bursa* |  |  | X |
|  | *Sufflamen chrysopterus* | X |  |  |
|  | Balistidae species A | X |  |  |
| Blenniidae | *Petroscirtes variabilis* |  | X |  |
|  | *Salarias alboguttatus* |  | X |  |
|  | *Salarias fasciatus* |  | X |  |
|  | *Salarias* species A | X |  |  |
| Caracanthidae | *Caracanthus maculatus* |  |  | X |
| Centriscidae | *Aeoliscus strigatus* | X | X |  |
| Chaetodontidae | *Chaetodon auriga* |  | X |  |
|  | *Chaetodon baronessa* | X |  |  |
|  | *Chaetodon bennetti* |  | X |  |
|  | *Chaetodon citrinellus* |  |  | X |
|  | *Chaetodon ephippium* | X | X |  |
|  | *Chaetodon lunulatus* | X | X |  |
|  | *Chaetodon octofascialis* | X |  |  |
|  | *Chaetodon plebeius* |  | X |  |
|  | *Chaetodon speculum* |  | X |  |
|  | *Chaetodon trifascialis* | X | X | X |
|  | *Chaetodon ulientensis* |  | X |  |
|  | *Chaetodon vagabundus* |  |  | X |
| Cirrhitidae | *Cirrhitichthys falco* | X |  |  |
|  | *Neocirrhitus armatus* |  |  | X |
|  | *Paracirrhitus arcatus* |  |  | X |
| Gobidae | *Paragobiodon echinocephalus* | X | X |  |
|  | *Paragobiodon melanosomus* | X |  |  |
|  | *Paragobiodon cf. xanthosomus* | X |  |  |
|  | *Paragobiodon xanthosomus* | X | X |  |
|  | *Amblygobius phaelaena* | X | X |  |
|  | *Asteropteryx semipuctatus* |  | X |  |
|  | *Asterropteryx ensifera* |  |  | X |
|  | *Atrosalarias fuscus holomelas* |  | X |  |
|  | *Coryphopterus neophytus* |  |  | X |
|  | *Ctenogobiops pomastictus* |  | X |  |
|  | *Eviota nigriventris* | X |  |  |
|  | *Eviota pellucida* | X |  |  |
|  | *Eviota queenslandica* |  | X |  |
|  | *Fusigobius duospilus* |  | X |  |
|  | *Gnatholepis anjerensis* |  | X | X |
|  | *Gobiodon albofasciatus* | X |  |  |
|  | *Gobiodon brochus* |  | X |  |
|  | *Gobiodon ceramicus* | X | X |  |
|  | *Gobiodon citrinus* |  | X |  |
|  | *Gobiodon erithrospirus* |  | X |  |
|  | *Gobiodon histrio* | X | X |  |
|  | *Gobiodon oculineatus* |  | X |  |
|  | *Gobiodon okinawae* | X | X |  |
|  | *Gobiodon okulolineatus* | X |  |  |
|  | *Gobiodon quinquestrigatus* | X | X |  |
|  | *Gobiodon* species C | X |  |  |
|  | *Gobiodon unicolor* |  | X |  |
|  | *Istigobius decoratus* | X | X |  |
|  | *Istigobius goldmanni* |  | X |  |
|  | *Istigobius rigilius* |  | X |  |
|  | *Pleurosicya micheli* |  | X |  |
|  | *Valencienna longipinnis* |  | X |  |
|  | *Valenciennea muralis* | X |  |  |
|  | *Valenciennea strigata* |  |  | X |
| Haemulidae | *Plectorhinchus lessonii* |  | X |  |
| Holocentridae | *Myripristis* species A | X |  |  |
|  | *Neoniphon sammara* |  | X | X |
|  | *Neoniphon* species A | X |  |  |
|  | *Sargocentron microstoma* |  |  | X |
|  | *Sargocentron* species A |  | X |  |
| Labridae | *Cheilinus chlorourus* |  | X | X |
|  | *Cheilinus trilobatus* |  | X |  |
|  | *Cirrhilabrus exquisitus* |  |  | X |
|  | *Coris batuensis* |  | X |  |
|  | *Halichoeres chloropterus* | X |  |  |
|  | *Halichoeres cyanopleura* | X |  |  |
|  | *Halichoeres melanurus* | X | X |  |
|  | *Halichoeres scapularis* | X |  |  |
|  | *Halichoeres trimaculatus* |  | X | X |
|  | *Hemigymnus fasciatus* | X |  |  |
|  | *Hemigymnus melapterus* |  | X |  |
|  | *Iniistius aneitensis* |  | X |  |
|  | *Labrichthys unilineatus* | X |  |  |
|  | *Novaculichthys taenioarus* |  |  | X |
|  | *Oxycheilinus bimaculatus* | X |  | X |
|  | *Pseudocheilinus evanidus* |  |  | X |
|  | *Pseudocheilinus hexataenia* | X |  | X |
|  | *Stethojulis bandanensis* |  |  | X |
|  | *Stethojulis strigiventer* |  | X |  |
|  | *Thalassoma lunare* | X | X |  |
|  | *Wetmorella* species A | X |  |  |
| Lethrinidae | *Gnathodentex cf. aureolineatus* | X |  |  |
|  | *Lethrinus olivaceus* |  |  | X |
|  | *Monotaxis grandoculis* | X |  |  |
| Lutjanidae | *Lutjanus carponatus* |  | X |  |
|  | *Lutjanus gibbus* | X | X |  |
|  | *Lutjanus kasmira* | X |  |  |
|  | *Lutjanus monostigma* | X | X |  |
| Microdesmidae | *Ptereleotris microlepis* | X |  |  |
| Monacanthidae | *Oxymonacanthus longirostris* |  | X |  |
|  | *Paracanthurus hepatus* | X | X |  |
|  | *Pervagor nigrolineatus* | X |  |  |
| Mullidae | *Mulloidichthys flavolineatus* |  | X |  |
|  | *Parupeneus barberinoides* | X |  |  |
|  | *Parupeneus barberinus* | X | X | X |
|  | *Parupeneus ciliatus* | X |  |  |
|  | *Parupeneus heptacanthus* | X |  |  |
|  | *Parupeneus insularis* |  |  | X |
|  | *Parupeneus macronemma* |  | X |  |
|  | *Parupeneus multifasciatus* |  |  | X |
|  | *Parupeneus pleurostigma* | X |  | X |
| Muraenidae | *Echidna nebulosa* |  |  | X |
| Nemipteridae | *Scolopsis affinis* | X |  |  |
|  | *Scolopsis* species A |  | X |  |
|  | *Scolopsis temporalis* | X |  |  |
|  | *Scolopsis* species B | X |  |  |
| Ostraciidae | *Ostracion cubicus* |  |  | X |
| Pinguipedidae | *Parapercis cylindrica* |  | X |  |
|  | *Parapercis millepunctata* |  |  | X |
| Pomacanthidae | *Centropyge bispinosus* |  |  | X |
| Pomacentridae | *Amblyglyphidodon curacao* |  | X |  |
|  | *Chromis viridis* | X | X | X |
|  | *Chrysiptera brownriggii* |  |  | X |
|  | *Dascyllus aruanus* | X | X | X |
|  | *Dascyllus flavicaudus* |  |  | X |
|  | *Dascyllus melanurus* | X |  |  |
|  | *Dascyllus reticulatus* | X | X |  |
|  | *Dascyllus trimaculatus* | X | X | X |
|  | *Dischistodus perspicillatus* | X | X |  |
|  | *Dischistodus prosopotaenia* |  | X |  |
|  | *Pomacentrus adelus* | X | X |  |
|  | *Pomacentrus amboinensis* | X | X |  |
|  | *Pomacentrus aurifrons* | X |  |  |
|  | *Pomacentrus chrysurus* |  | X |  |
|  | *Pomacentrus coelestis* | X |  |  |
|  | *Pomacentrus grammorhynchus* |  | X |  |
|  | *Pomacentrus moluccensis* |  | X |  |
|  | *Pomacentrus nagasakiensis* |  | X |  |
|  | *Pomacentrus pavo* | X | X | X |
|  | *Pomacentrus simsiang* | X |  |  |
|  | *Premnas biaculeatus* | X |  |  |
|  | *Pseudochromis flammicauda* |  | X |  |
|  | *Pseudochromis fuscus* | X | X |  |
|  | *Pseudochromis marshallensis* |  | X |  |
|  | *Stegastes lividus* | X | X |  |
|  | *Stegastes nigricans* |  |  | X |
| Pseudochromidae | *Cypho purpurascens* |  | X |  |
|  | *Ogilbyina queenslandiae* |  | X |  |
| Scaridae | *Chlorurus sordidus* |  |  | X |
|  | *Scaridae species A* | X | X |  |
|  | *Scaridae species B* | X | X |  |
|  | *Scarus psittacus* |  |  | X |
| Scorpaenidae | *Pterois species A* | X | X |  |
|  | *Pterois antennata* |  |  | X |
|  | *Pterois volitans* |  |  | X |
|  | Scorpaenidae species A | X | X |  |
| Serranidae | *Cephalopholis boenak* | X |  |  |
|  | *Cephalopholis cyanostigma* | X |  |  |
|  | *Cephalopholis microprion* | X |  |  |
|  | *Cephalopholis urodeta* | X |  |  |
|  | *Cromileptes altivelis* |  | X |  |
|  | *Epinephelus maculatus* | X | X |  |
|  | *Epinephelus ongus* | X |  |  |
|  | *Epinephelus quoyanus* |  | X |  |
| Siganidae | *Siganus doliatus* |  | X |  |
|  | *Siganus puellus* |  | X |  |
|  | *Siganus punctatus* |  | X |  |
| Syngnathidae | *Corythoichthys intestinalis* | X |  |  |
|  | *Micrognathus andersonii* |  | X |  |
|  | *Synodus dermatogenys* |  |  | X |
| Tetraodontidae | *Arothron meleagris* | X |  |  |
|  | *Canthigaster bennetti* | X | X | X |
|  | *Canthigaster papua* | X |  |  |
|  | *Canthigaster valentini* | X | X |  |
| Zanclidae | *Zanclus cornutus* |  |  | X |
