# Supplementary material for: Reef Fishes in Biodiversity Hotspots Are at Greatest Risk from Loss of Coral Species
Source: PLoS One. 2015 May 13;10(5):e0124054. doi: 10.1371/journal.pone.0124054 (PMC4430502; doi:10.1371/journal.pone.0124054)
Supplement: S2 Table — The CAP analysis examining the fish communities present on each of the 45 1 m2 experimental plots captured a large amount of the variation in community structure in the first two components, with the two primary axes (CAP 1 and CAP 2) accounting for 43% of the total variance. Only those species with loadings scores < -0.2 or > 0.2 on at least one of the two axes (29 out of 57 species observed) are presented. (DOCX) [file pone.0124054.s002.docx]

**S2 Table.** Species loading scores obtained from a Canonical Analysis of Principal Coordinates (CAP) ordination plot constructed on a Bray-Curtis dissimilarity matrix of log-transformed fish abundance data collected from Moorea, French Polynesia. The CAP analysis examining the fish communities present on each of the 45 1 m^2^ experimental plots captured a large amount of the variation in community structure in the first two components, with the two primary axes (CAP 1 and CAP 2) accounting for 43% of the total variance. Only those species with loadings scores < -0.2 or > 0.2 on at least one of the two axes (29 out of 57 species observed) are presented.

| Family | Genus species | CAP Axis 1 | CAP Axis 2 |
| --- | --- | --- | --- |
| Acanthuridae | *Acanthurus nigricauda* | -0.4241 | -0.1621 |
|  | *Ctenochaetus binotatus* | 0.0510 | 0.4024 |
|  | *Naso literatus* | -0.2335 | -0.1565 |
|  | *Naso unicornis* | -0.2232 | -0.1342 |
|  | *Zebrasoma scopas* | -0.0951 | -0.2721 |
| Apogonidae | *Ostorhinchus nigrofasciatus* | 0.1661 | -0.2038 |
| Caracanthidae | *Caracanthus maculatus* | -0.1616 | -0.2991 |
| Chaetodontidae | *Chaetodon citrinellus* | -0.1127 | -0.4102 |
| Cirrhitidae | *Neocirrhitus armatus* | -0.1166 | -0.2846 |
| Gobiidae | *Gnatholepis anjerensis* | 0.2199 | 0.0799 |
|  | *Valenciennea strigata* | -0.0186 | 0.2573 |
| Holocentridae | *Neoniphon sammara* | -0.2249 | -0.1764 |
| Labridae | *Cheilinus chlorourus* | -0.2759 | -0.2828 |
|  | *Halichoeres trimaculatus* | 0.3295 | 0.2822 |
|  | *Novaculichthys taenioarus* | -0.2307 | 0.3696 |
|  | *Oxycheilinus bimaculatus* | 0.0314 | 0.5121 |
|  | *Stethojulis bandanensis* | -0.1050 | -0.3583 |
| Lutjanidae | *Lethrinus olivaceus* | 0.2005 | 0.0147 |
| Mullidae | *Parupeneus multifasciatus* | -0.4308 | -0.3453 |
| Mullidae | *Parupeneus pleurostigma* | -0.3611 | -0.2641 |
| Pomacanthidae | *Centropyge bispinosus* | -0.1537 | 0.2947 |
| Pomacentridae | *Chromis viridis* | -0.1049 | -0.5682 |
|  | *Dascyllus aruanus* | -0.0490 | -0.4127 |
|  | *Dascyllus flavicaudus* | -0.0381 | -0.7564 |
|  | *Dascyllus trimaculatus* | -0.2111 | -0.0383 |
|  | *Pomacentrus pavo* | -0.5352 | 0.3006 |
|  | *Stegastes nigricans* | -0.2776 | -0.0356 |
| Scaridae | *Chlorurus sordidus* | -0.2630 | -0.2480 |
| Tetraodontidae | *Canthigaster bennetti* | -0.0943 | -0.2536 |
