# Supplementary material for: Reef Fishes in Biodiversity Hotspots Are at Greatest Risk from Loss of Coral Species
Source: PLoS One. 2015 May 13;10(5):e0124054. doi: 10.1371/journal.pone.0124054 (PMC4430502; doi:10.1371/journal.pone.0124054)
Supplement: S3 Table — The CAP analysis examining the fish communities present on each of the 45 1 m2 experimental plots captured a large amount of the variation in community structure in the first two components, with the two primary axes (CAP 1 and CAP 2) accounting for 34% of the total variance. Only those species with loadings scores < -0.2 or > 0.2 on at least one of the two axes (65 out of 107 species observed) are presented. (DOCX) [file pone.0124054.s003.docx]

**S3 Table.** Species loading scores obtained from a Canonical Analysis of Principal Coordinates (CAP) ordination plot constructed on a Bray-Curtis dissimilarity matrix of log-transformed fish abundance data collected from Lizard Island, Great Barrier Reef, Australia. The CAP analysis examining the fish communities present on each of the 45 1 m^2^ experimental plots captured a large amount of the variation in community structure in the first two components, with the two primary axes (CAP 1 and CAP 2) accounting for 34% of the total variance. Only those species with loadings scores < -0.2 or > 0.2 on at least one of the two axes (65 out of 107 species observed) are presented.

| Family | Genus species | CAP Axis 1 | CAP Axis 2 |
| --- | --- | --- | --- |
| Acanthuridae | Acanthuridae species A | -0.3825 | 0.0993 |
|  | *Paracanthurus hepatus* | -0.1616 | -0.2618 |
| Apogonidae | *Apgon leptacanthus* | -0.2785 | 0.0706 |
|  | *Apogon cf. exostigma* | -0.2969 | -0.1635 |
|  | *Apogon thermalis* | 0.1848 | 0.2992 |
|  | *Apogon ventrifasciatus* | 0.0412 | 0.2616 |
|  | *Ostorhinchus cyanosoma* | 0.1694 | 0.4070 |
| Blenniidae | *Salarias fasciatus* | 0.2242 | -0.0774 |
| Chaetodontidae | *Chaetodon ephippium* | -0.2774 | 0.1574 |
|  | *Chaetodon lunulatus* | -0.2333 | 0.0943 |
|  | *Chaetodon plebeius* | -0.3302 | 0.3945 |
|  | *Chaetodon trifascialis* | 0.2347 | -0.0097 |
| Gobiidae | *Amblygobius phaelaena* | 0.2155 | -0.3078 |
|  | *Asteropteryx semipuctatus* | -0.2003 | -0.0983 |
|  | *Ctenogobiops pomastictus* | 0.3464 | -0.1647 |
|  | *Eviota queenslandica* | 0.2594 | -0.1560 |
|  | *Fusigobius duospilus* | 0.2977 | -0.0748 |
|  | *Gobiodon brochus* | -0.2016 | -0.3572 |
|  | *Gobiodon ceramicus* | -0.3695 | -0.1112 |
|  | *Gobiodon citrinus* | 0.5414 | -0.3184 |
|  | *Gobiodon erithrospirus* | -0.1616 | -0.2618 |
|  | *Gobiodon histrio* | -0.2795 | -0.1661 |
|  | *Gobiodon okinawae* | 0.3471 | 0.2549 |
|  | *Gobiodon quinquestrigatus* | -0.3103 | -0.2506 |
|  | *Istigobius rigilius* | 0.0600 | 0.2275 |
|  | *Paragobiodon echinocephalus* | -0.2045 | 0.3446 |
|  | *Paragobiodon xanthosomus* | -0.6054 | -0.0581 |
|  | *Pleurosicya micheli* | 0.2333 | -0.0746 |
| Haemulidae | *Plectorhinchus lessonii* | -0.1168 | -0.2275 |
| Holocentridae | *Neoniphon sammara* | -0.2403 | 0.0866 |
| Labridae | *Cheilinus chlorourus* | -0.2357 | -0.1700 |
|  | *Choris batuensis* | 0.3698 | -0.2507 |
|  | *Halichoeres trimaculatus* | -0.0894 | -0.2276 |
|  | *Iniistius aneitensis* | 0.2450 | -0.2197 |
|  | *Thalassoma lunare* | -0.3017 | 0.2295 |
| Lutjanidae | *Lutjanus carponatus* | 0.2773 | -0.2410 |
|  | *Lutjanus gibbus* | -0.0681 | 0.5430 |
|  | *Lutjanus monostigma* | 0.0108 | 0.2275 |
| Mullidae | *Parupeneus barberinus* | -0.2712 | -0.0346 |
|  | *Parupeneus macronemma* | -0.0505 | -0.3411 |
| Nemipteridae | *Scolopsis* species A | -0.1616 | -0.2618 |
| Pomacentridae | *Amblyglyphidodon curacao* | 0.2376 | 0.3002 |
|  | *Chromis viridis* | -0.6617 | -0.3812 |
|  | *Dascyllus aruanus* | -0.6290 | 0.5761 |
|  | *Dascyllus reticulatus* | -0.3007 | 0.4554 |
|  | *Dascyllus trimaculatus* | -0.2019 | -0.0610 |
|  | *Dischistodus prosopotaenia* | -0.0600 | -0.3009 |
|  | *Pomacentrus adelus* | -0.2166 | -0.1015 |
|  | *Pomacentrus amboinensis* | -0.2350 | 0.4692 |
|  | *Pomacentrus grammorhynchus* | -0.2673 | -0.1823 |
|  | *Pomacentrus moluccensis* | -0.4743 | 0.3339 |
|  | *Pomacentrus nagasakiensis* | -0.2367 | 0.1722 |
|  | *Pomacentrus pavo* | -0.2982 | 0.3014 |
|  | *Stegastes lividus* | -0.3338 | -0.2560 |
| Pseudochromidae | *Cypho purpurascens* | 0.0416 | 0.2700 |
|  | *Ogilbyina queenslandiae* | -0.1114 | -0.3643 |
|  | *Pseudochromis fuscus* | -0.0681 | -0.4015 |
| Scaridae | Scaridae species A | -0.3731 | -0.2715 |
|  | Scaridae species B | 0.3390 | -0.3483 |
| Scorpaenidae | *Scorpionfish* | -0.1310 | -0.2116 |
| Serranidae | *Cromileptes altivelis* | 0.0416 | 0.2700 |
|  | *Epinephelus maculatus* | -0.0497 | -0.2436 |
| Siganidae | *Siganus puellus* | -0.2634 | -0.1546 |
|  | *Siganus punctatus* | 0.0909 | 0.3002 |
| Tetraodontidae | *Canthigaster bennetti* | 0.2087 | -0.3797 |
