# Supplementary material for: Reef Fishes in Biodiversity Hotspots Are at Greatest Risk from Loss of Coral Species
Source: PLoS One. 2015 May 13;10(5):e0124054. doi: 10.1371/journal.pone.0124054 (PMC4430502; doi:10.1371/journal.pone.0124054)
Supplement: S4 Table — The CAP analysis examining the fish communities present on each of the 45 1 m2 experimental plots captured a large amount of the variation in community structure in the first two components, with the two primary axes (CAP 1 and CAP 2) accounting for 30% of the total variance. Only those species with loadings scores < -0.2 or > 0.2 on at least one of the two axes (59 out of 99 species observed) are presented. (DOCX) [file pone.0124054.s004.docx]

**S4 Table.** Species loading scores obtained from a Canonical Analysis of Principal Coordinates (CAP) ordination plot constructed on a Bray-Curtis dissimilarity matrix of log-transformed fish abundance data collected from Kimbe Bay, Papua New Guinea. The CAP analysis examining the fish communities present on each of the 45 1 m^2^ experimental plots captured a large amount of the variation in community structure in the first two components, with the two primary axes (CAP 1 and CAP 2) accounting for 30% of the total variance. Only those species with loadings scores < -0.2 or > 0.2 on at least one of the two axes (59 out of 99 species observed) are presented.

| Family | Genus species | CAP Axis 1 | CAP Axis 2 |
| --- | --- | --- | --- |
| Acanthuridae | Acanthuridae species A | -0.2954 | 0.1585 |
|  | *Paracanthurus hepatus* | -0.4432 | -0.0484 |
| Apogonidae | *Apogon cyanosoma* | 0.1720 | -0.3280 |
|  | *Apogon hartzfeldii* | -0.0865 | -0.2276 |
| Balistidae | *Balistapus undulatus* | -0.2120 | 0.1990 |
|  | *Pseudobalistes flavimarginatus* | 0.2149 | -0.2631 |
|  | *Rhinecanthus verrucosus* | 0.3092 | -0.1439 |
|  | Balistidae species A | -0.1639 | -0.2421 |
| Blenniidae | *Salarias* species A | -0.2808 | -0.0588 |
| Chaetodontidae | *Chaetodon baronessa* | -0.6149 | -0.1927 |
|  | *Chaetodon lunulatus* | -0.3919 | -0.0453 |
|  | *Chaetodon octofascialis* | -0.4328 | -0.0300 |
|  | *Chaetodon trifascialis* | -0.2380 | -0.0451 |
| Cirrhitidae | *Cirrhitichthys falco* | -0.3798 | -0.0005 |
| Gobiidae | *Eviota nigriventris* | 0.1964 | 0.2109 |
|  | *Gobiodon albofasciatus* | -0.2731 | -0.2336 |
|  | *Gobiodon ceramicus* | -0.4713 | 0.1346 |
|  | *Gobiodon histrio* | -0.5724 | 0.1291 |
|  | *Gobiodon okulolineatus* | 0.0588 | 0.4727 |
|  | *Gobiodon quinquestrigatus* | -0.7080 | -0.0918 |
|  | *Gobiodon* species C | 0.2081 | 0.4849 |
|  | *Istigobius decoratus* | 0.1846 | -0.2213 |
|  | *Paragobiodon echinocephalus* | 0.3980 | 0.5770 |
|  | *Paragobiodon melanosomus* | -0.0863 | 0.2106 |
|  | *Paragobiodon xanthosomus* | -0.6397 | 0.1304 |
| Holocentridae | *Neoniphon* species A | -0.3537 | -0.2148 |
| Labridae | *Halichoeres cyanopleura* | -0.0479 | 0.2620 |
|  | *Halichoeres scapularis* | 0.2688 | -0.1936 |
|  | *Hemigymnus fasciatus* | -0.2380 | -0.0451 |
|  | *Oxycheilinus bimaculatus* | -0.1568 | -0.2268 |
|  | *Pseudocheilinus hexataenia* | -0.2249 | 0.1464 |
|  | *Wetmorella* species A | 0.1846 | -0.2213 |
| Lethrinidae | *Monotaxis grandoculis* | -0.0549 | -0.4525 |
| Lutjanidae | *Lutjanus monostigma* | -0.1020 | -0.4019 |
| Monacanthidae | *Pervagor nigrolineatus* | -0.0013 | 0.2034 |
| Mullidae | *Parupeneus barberinus* | -0.1666 | -0.2226 |
|  | *Parupeneus ciliatus* | 0.0560 | -0.2318 |
|  | *Parupeneus pleurostigma* | -0.0039 | -0.2299 |
| Nemipteridae | *Scolopsis affinis* | 0.2055 | -0.3033 |
|  | *Scolopsis* species B | -0.0248 | 0.2172 |
| Pomacentridae | *Chromis viridis* | -0.5885 | 0.0591 |
|  | *Dascyllus aruanus* | 0.2073 | -0.0144 |
|  | *Dascyllus melanurus* | -0.3972 | 0.2913 |
|  | *Dascyllus reticulatus* | -0.2220 | 0.4397 |
|  | *Dascyllus trimaculatus* | -0.0080 | 0.2317 |
|  | *Dischistodus perspicillatus* | 0.2278 | -0.1618 |
|  | *Pomacentrus amboinensis* | -0.0435 | 0.2361 |
|  | *Pomacentrus pavo* | 0.0578 | 0.3016 |
|  | *Premnas biaculeatus* | -0.0479 | 0.2620 |
|  | *Stegastes lividus* | -0.2883 | 0.0904 |
| Scaridae | Scaridae species A | -0.3282 | -0.0076 |
|  | Scaridae species B | 0.0572 | -0.2368 |
| Scorpaenidae | *Pterois* species A | -0.0687 | -0.3483 |
| Serranidae | *Cephalopholis boenak* | 0.1846 | -0.2213 |
|  | *Cephalopholis microprion* | -0.0106 | 0.3761 |
|  | *Epinephelus maculatus* | -0.0165 | -0.2205 |
| Tetraodontidae | *Canthigaster bennetti* | 0.2488 | -0.2563 |
|  | *Canthigaster papua* | -0.2302 | -0.1080 |
|  | *Canthigaster valentini* | 0.1822 | -0.2337 |
