# Supplementary material for: Reef Fishes in Biodiversity Hotspots Are at Greatest Risk from Loss of Coral Species
Source: PLoS One. 2015 May 13;10(5):e0124054. doi: 10.1371/journal.pone.0124054 (PMC4430502; doi:10.1371/journal.pone.0124054)

**S1 Figure.** Total number of fish species per experimental patch reef on each coral diversity treatment at the final census at the three study sites. Data are the mean (± 1 SE) number of fish species per plot. Lines represent linear regressions fitted to the individual plot data, and the slopes of these lines provide an estimate of the sensitivity of fish species richness to changes in coral diversity for each location (see Figure 3); Moorea: F_1,43_ = 1.77; P = 0.19; Lizard: F_1,43_ = 2.41; P = 0.13; Kimbe: F_1,43_ = 14.18; P < 0.001. N = 30 patch reefs per geographic location for coral diversity of 1 species, N = 10 patch reefs per geographic location for coral diversity of 3 species, and N = 5 patch reefs per geographic location for coral diversity of 6 species.


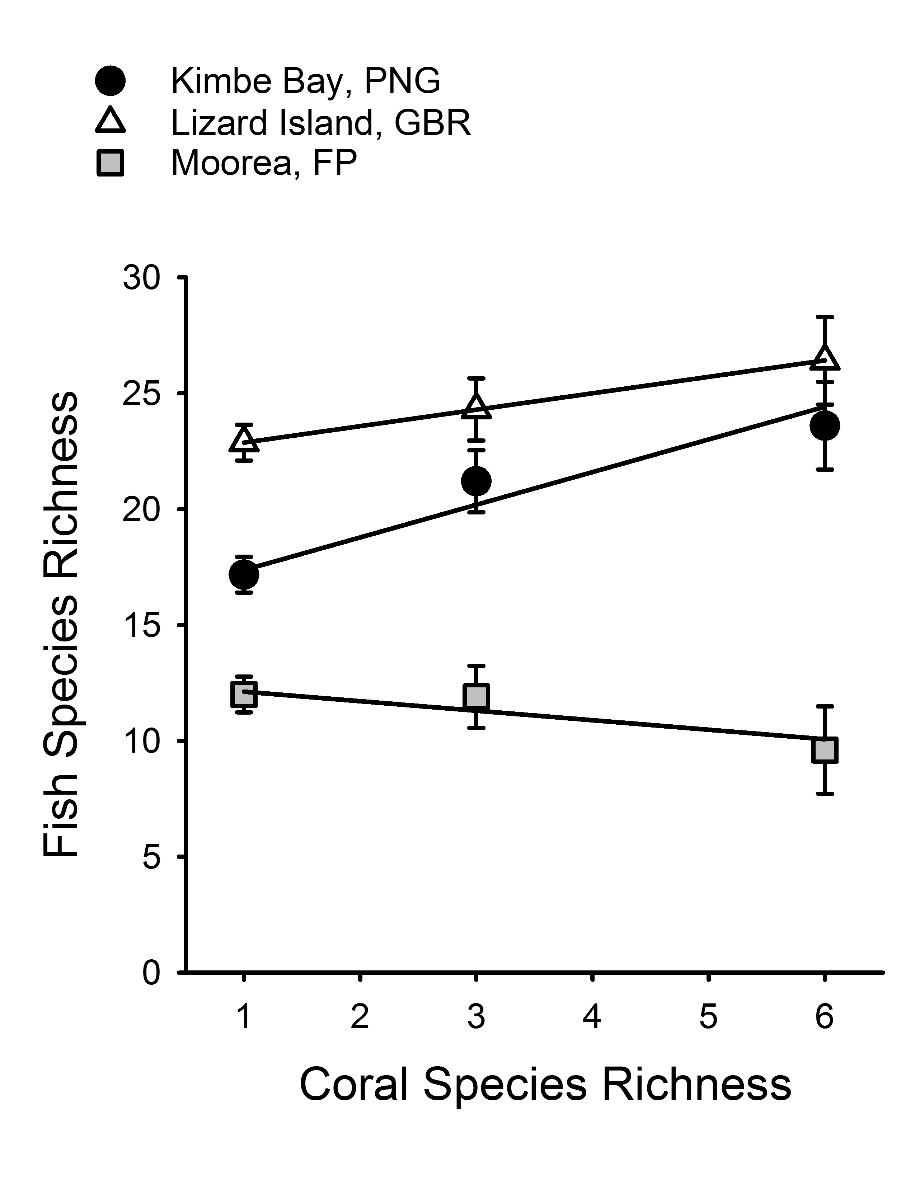

Supplement: S1 Fig — Data are the mean (± 1 SE) number of fish species per plot. Lines represent linear regressions fitted to the individual plot data, and the slopes of these lines provide an estimate of the sensitivity of fish species richness to changes in coral diversity for each location (see Fig 4); Moorea: F1,43 = 1.77; P = 0.19; Lizard: F1,43 = 2.41; P = 0.13; Kimbe: F1,43 = 14.18; P < 0.001. N = 30 patch reefs per geographic location for coral diversity of 1 species, N = 10 patch reefs per geographic location for coral diversity of 3 species, and N = 5 patch reefs per geographic location for coral diversity of 6 species. (DOCX) [file pone.0124054.s005.docx]
